# Pavlovian influences on learning differ between rats and mice in a counter-balanced Go/NoGo judgement bias task

**DOI:** 10.1016/j.bbr.2017.05.044

**Published:** 2017-07-28

**Authors:** Samantha Jones, Elizabeth S. Paul, Peter Dayan, Emma S.J. Robinson, Michael Mendl

**Affiliations:** a Centre for Behavioural Biology, School of Veterinary Science, University of Bristol, UK; b Gatsby Computational Neuroscience Unit, University College London, UK; c School of Physiology, Pharmacology and Neuroscience, University of Bristol, UK

**Keywords:** Rat, Mouse, Cognitive bias, Learning, Pavlovian control, Go/NoGo

## Abstract

Judgement bias tests of animal affect and hence welfare assume that the animal’s responses to ambiguous stimuli, which may herald positive or negative outcomes, are under instrumental control and reflect ‘optimism’ or ‘pessimism’ about what will happen. However, Pavlovian control favours responses (e.g. approach or withdrawal) according to the valence associated with a stimulus, rather than the anticipated response outcomes. Typically, positive contexts promote action and approach whilst negative contexts promote inhibition or withdrawal. The prevalence of Go-for-reward (*Go-pos*) and NoGo-to-avoid-punishment (*NoGo-neg*) judgement bias tasks reflects this Pavlovian influence. A Pavlovian increase or decrease in activity or vigour has also been argued to accompany positive or negative affective states, and this may interfere with instrumental Go or NoGo decisions under ambiguity based on anticipated decision outcomes. One approach to these issues is to develop counter-balanced *Go-pos*/*NoGo-neg* and *Go-neg*/*NoGo-pos* tasks. Here we implement such tasks in Sprague Dawley rats and C57BL/6J mice using food and air-puff as decision outcomes. We find striking species/strain differences with rats achieving criterion performance on the *Go-pos/NoGo-neg* task but failing to learn the *Go-neg*/*NoGo-pos* task, in line with predictions, whilst mice do exactly the opposite. Pavlovian predispositions may thus differ between species, for example reflecting foraging and predation ecology and/or baseline activity rates. Learning failures are restricted to cues predicting a negative outcome; use of a more powerful air-puff stimulus may thus allow implementation of a fully counter-balanced task. Rats and mice achieve criterion faster than in comparable automated tasks and also show the expected generalisation of responses across ambiguous tones. A fully counter-balanced task thus offers a potentially rapidly implemented and automated method for assessing animal welfare, identifying welfare problems and areas for welfare improvement and 3Rs *Refinement*, and assessing the effectiveness of refinements.

## Highlights

• We develop an automated counter-balanced Go/NoGo judgement bias task for rodents.
• Rats learn Go-for-reward/NoGo-to-avoid-punishment but not the reverse contingency.
• Mice do exactly the opposite; species may differ in Pavlovian control of decisions.
• Foraging, predator avoidance, and baseline activity may influence Pavlovian control.
• Both species show the predicted generalisation of responses in ambiguity tests.

## 1 Introduction

Valid translational models of affective disorders, better measures of animal welfare that allow more effective detection of welfare problems and implementation of 3Rs *Refinements*, and a deeper understanding of the evolutionary history and mechanistic underpinnings of affective states, all require accurate measurement of affect in animals. Over the last decade, an assay to measure decision-making under ambiguity (the so-called ‘cognitive’ or ‘judgement’ bias test) has been used in a wide range of species as a new indicator of affective valence (positivity or negativity) [1], [2], [3], [4], [5], [6], [7], [8]. This approach is based on empirical findings that people in negative affective states (as judged by their reports of the subjective experience of negative emotions) make more negative and pessimistic judgements about ambiguous or future events than happier people [9], [10], and on theoretical arguments that such affect-related changes in decisions about ambiguity have adaptive value and hence are likely to be observed across species [11], [12], [13]. For example, a negative state resulting from recent experience of negative or punishing events should increase prior expectations of the future likelihood of punishment, thus favouring cautious decisions, especially under ambiguity where there is a lack of information about the true current situation, including choice outcomes [12].

Originally developed for rats [1], the generic judgement bias assay involves training animals to make one type of response (*P*: e.g. lever-press) to a cue predicting a positive event or reward (*p*: e.g. a tone of a particular frequency) in order to receive that reward (e.g. food delivery), and another type of response (*N*: e.g. no lever-press) to a cue predicting a negative event or punisher (*n*: e.g. a tone of a different frequency) in order to avoid that event (e.g. white noise). Once this discrimination is learnt, subjects receive occasional ‘ambiguous’ cues (e.g. tones in between *p* and *n*). Their tendency to make *P* (‘optimistic’) or *N* (‘pessimistic’) responses to these ambiguous cues is used to infer whether they anticipate that a positive or negative outcome is more likely, and hence whether their underlying affective state is, respectively, relatively positive or negative. The task has been adapted for use in a range of mammals (e.g. rats [14], [15], [16], [17], [18], [19], [20]; mice [21], [22]; hamsters [23]; dogs [24], [25]; sheep [26], [27]; pigs [28], [29]; cattle [30], [31]; monkeys [32], [33]; peccary [34]), birds (e.g. starling [35], [36]; chicken [37], [38]), and insects [39], [40], [41], and it has also been back-translated to humans [42], [43], [44], [45]. A variety of affect manipulations has been employed. Many of the published findings (but not all [37], [46], [47], [48], [49], [50], [51], [52]) are consistent with the hypothesis that, like humans, non-human animals in assumed negative affective states show negatively biased judgements of ambiguity. Thus, judgement biases may be useful indicators of the valence of an animal’s affective state even though, like all measures of animal affect, they cannot tell us whether the inferred affective state is consciously experienced in other species [53].

Judgement bias tests assume that the animal’s response to an ambiguous stimulus is under instrumental control; it reflects the learnt contingency between response and outcome (e.g. response *P* indicates anticipation of a positive outcome). However, decisions are also influenced by Pavlovian control which elicits responses, primarily approach or withdrawal, according to the valence associated with a cue rather than the consequences of the responses. There is evidence for a natural predisposition for active approach and engagement in a rewarding context (e.g. in response to a *p* cue that may be intrinsically rewarding, or acquire positive valence through a rewarding outcome), and inhibition or withdrawal in the face of punishment (e.g. in response to an *n* cue) [54], [55], [56], [57]. The resulting ‘hard-wired’ stimulus-response decision policies may be implemented in the functional architecture of the basal ganglia where excitation of the ‘direct pathway’ generates active responses for reward whereas excitation of the ‘indirect pathway’ inhibits motor responses in the context of punishment. Likewise, the dopaminergic system plays a role in active reward-seeking behaviour whilst the serotonergic system may be more involved in aversion-related behavioural inhibition [54], [58], [59].

One effect of this Pavlovian influence is that active *P* responses are learnt faster than active *N* responses. For example, in balanced active two-choice judgement bias tasks (e.g. *P* = left lever press; *N* = right lever press), active lever-pressing to avoid a predicted punisher (*N*) is much more difficult to learn than the same response to acquire a predicted reward (*P*) (e.g. 6 vs 13–17 days; 14–17 vs 25–26 days; see Ref. [19]), making these tasks very time consuming to implement. A pragmatic, and likely implicit, consequence is the popularity of making the *P* response active (Go) and the *N* response inactive (NoGo) in the majority of judgement bias tasks.

However, in these commonly-used Go-for-reward and NoGo-to-avoid-punishment tasks, Pavlovian influences can further complicate interpretation of *P* or *N* responses to ambiguity during an affective manipulation. This is because the relative ease of performing *P* and *N* may be directly influenced by affective state, hence obscuring the ability of affect to modulate judgement of ambiguity. In particular, an increase or decrease in vigour has been argued to accompany positive or negative affective states respectively [59], hence influencing the type of decision response shown (Go or NoGo) irrespective of the associated outcome of that decision. Experimental treatments may also cause non-affect related changes in general activity that favour Go or NoGo responses, and any extinction of response to ambiguous cues [60], or failure to attend when a cue is presented, will lead to a NoGo response that may be erroneously interpreted as ‘pessimistic’.

One hitherto unexplored solution to these problems is to counter-balance the relationship between vigour and valence, using both a Go-for-reward vs NoGo-to-avoid-punishment contingency as well as its opposite (NoGo-for-reward vs Go-to-avoid-punishment). This design has been employed to examine Pavlovian biases in human studies [55], [58], [61], [62], but has not been used in the context of animal tests of judgement bias. Such a counter-balanced task would allow direct investigation of the interplay between affective valence, outcome prediction (‘pessimism’ vs ‘optimism’), and Pavlovian response selection (active (Go) vs inactive (NoGo)). For example, if positive valence generates both ‘optimism’ and enhanced vigour, positive (‘optimistic’) choices under ambiguity would be clearly evident in the Go-for-reward contingency but less so under NoGo-for-reward where the two effects are in opposition. If ‘optimistic’ responses are clearly seen in both contingencies, this would indicate that Pavlovian control of response selection is subservient to instrumental control [54]. Thus, a counter-balanced task has the potential to shed new light on processes mediating decision-making under ambiguity in the judgement bias task.

The aim of this study was, therefore, to investigate how easy it is for laboratory rodents to learn counter-balanced Go/NoGo tasks and, in particular, to investigate the hypothesis that Pavlovian predispositions favour more rapid learning of the Go-for-reward/NoGo-to-avoid-punishment contingency. We also sought to develop automated methods which can be readily implemented using widely available equipment, and to develop tests for both rats and mice. The latter are relatively understudied in cognitive bias research, and automated testing is notably absent. The development of an easily implementable test would allow more widespread use of this measure of laboratory animal affect and welfare, hence facilitating better detection of animal welfare problems and areas where 3Rs *Refinement* of housing or experimental procedures would improve welfare, and more accurate assessment of the effectiveness of refinements. To these ends, we studied commonly used rat (Sprague Dawley) and mouse (C57BL/6J) strains in a shuttle-box task in which subjects were trained on one of the two Go/NoGo contingencies. For example, in the Go-for-reward/NoGo-to-avoid-punishment contingency, subjects needed to respond to cues predicting reward by shuttling (Go) from the half of the box in which they were currently located to the other half in order to receive reward. In contrast, they had to respond to negative cues by staying (NoGo) in their current half of the box in order to avoid a negative event.

## 2 Materials and methods

### 2.1 Experiment 1: rat study

#### 2.1.1 Animals and husbandry

The experimental subjects were 12 male Sprague Dawley rats (*Rattus norvegicus;* Harlan UK Ltd, UK). They were 3 months-old on arrival, and housed in pairs in standard cages (56 cm L × 33.5 cm W × 20 cm H, containing sawdust, shredded bedding, red shelter, wooden chew block and cardboard tube), under a 12hr reversed light-dark cycle (lights on 1900-0700). Food (LabDiet, St Louis, MO, USA) and water were available *ad-libitum.* All the rats were checked regularly for any health issues throughout the experiment, which was conducted under UK Home Office licence 30/2954.

#### 2.1.2 Apparatus

**Fig. 1:**
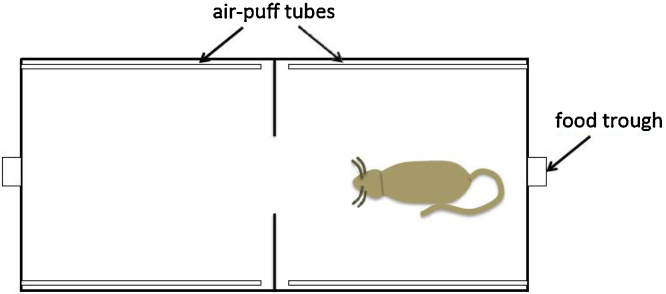
Plan view of the shuttle box apparatus. In each half of the apparatus, food was dispensed at the food troughs and air-puffs were delivered through tubes located on the side walls as shown. Rats and mice were trained to either shuttle (Go) from their current location when they heard the tone predicting food in order to collect the food from the opposite chamber (*Go-pos*) or to stay (NoGo) in their current location when they heard the tone predicting air puff in order to avoid delivery of air puff in the other chamber (*NoGo-neg*), or they were trained on the opposite contingency (*NoGo-pos/Go-neg*).

Two shuttle boxes (50.8 cm L × 25.4 cm W × 30.5 cm H) and associated hardware were used. Each box was divided in half by a metal panel to form two chambers between which the rats could move (shuttle) through a central opening (8 cm W × 9 cm H) in the panel (Fig. 1). This apparatus allowed us to train the Go-(shuttle)-for-reward/NoGo-(stay)-to avoid-punishment and reverse contingencies. Sensors monitored rat movement between the chambers. A loudspeaker was positioned centrally above the panel separating the two compartments, and a feeding trough supplied by an automated food dispenser was positioned at each end of the shuttle box accessible through an opening (3.2 cm W × 4 cm H) in the rear wall of each chamber. The two food dispensers delivered Bioserv (Flemington, NJ, USA) Dustless Precision Pellets (45 mg sucrose pellets). Air-puffs could also be delivered into each chamber using apparatus based on [63]. One piece of copper tubing (3 mm internal diameter and total length 34.5 cm (length inside chamber 24.5 cm)) was attached to each of the two side walls of each chamber. The tubes were perforated with nine holes × 2 mm diameter and attached to the walls via plastic adhesive clips. Tygon tubing and plastic Y connectors were used to attach the tubing to olfactory control units (containing a solenoid rated to 50psi) and to the compressed air cylinder (BOC UK). Each shuttle box was placed inside a sound-proofed chamber (external 126 cm L × 100 cm W × 63 cm H; internal: 110 cm L × 85 cm W × 50.5 cm H) to allow simultaneous testing without sound spill-over between the two sets of equipment.

All hardware was manufactured by Coulbourn Instruments (Allentown, PA, USA) and operated by their Graphic State (v3.02) software. Protocols were written using Graphic State notation and used to control all behavioural tests. Necessary hardware (e.g. computer, keyboard, VDU, computer-hardware interface, audio signal generators) were located outside the testing room to allow the researcher to control the equipment and work under white light without disturbing the animals.

#### 2.1.3 Training and testing

During the first two weeks of the study, the animals were regularly handled and habituated to the shuttle boxes and the holding cages used to move them between housing and testing rooms. All training and testing was conducted in the dark, and between 0900 and 1700 on five days per week (Mon-Fri) in a testing room separate to that in which the animals lived.

##### 2.1.3.1 Positive tone training phase

All rats were first trained to respond to a cue (10 s tone) that predicted a positive outcome (one sucrose pellet), and were allocated to two different training contingency groups as follows. Six rats (one randomly selected from each cage) were trained to shuttle from one chamber to the other when the ‘positive’ tone (2 kHz at 76 dB or 8 kHz at 65 dB (cf. [64]), balanced across rats) sounded in order to acquire the reward (Go-pos), whilst the other six were trained to stay in their current chamber when the tone sounded in order to get the reward (NoGo-pos). Each session started with a 120 s inter-trial-interval (ITI), followed by the first trial which commenced with the tone being presented for 10s. For ‘Go-pos’ rats, shuttling from their current compartment during the tone resulted in a food pellet being delivered into the trough of the chamber which they had entered, and the tone stopping. If they did not shuttle before the 10 s tone ended, they did not receive a food pellet. For ‘NoGo-pos’ rats, a food pellet was delivered into the trough of their current compartment if they stayed in that compartment (didn’t shuttle) for the 10 s duration of the tone. If they shuttled to the other compartment during the tone, the tone stopped and they did not receive a pellet. If rats responded correctly they received an ITI of 105–115 s before the next trial began. If they responded incorrectly they received an ITI of 115–125s. Each trial started in whichever side of the shuttle box the rat was in at the time of tone onset. Rats received 15 trials per session. During the first session, the researcher guided rats to make the correct response when the tone sounded by either gently moving ‘Go’ rats through the opening into the adjacent chamber, or blocking the opening to prevent ‘NoGo’ rats from moving through it. For the first session six rats were tested per day, but from session two onwards all twelve rats were tested once a day, five days a week. Rats were tested in the same order each day and individuals from the same cage were tested at the same time. Once the rats were performing correctly on more than 70% of trials per session across two consecutive days, they entered the next phase of training.

##### 2.1.3.2 Negative tone training phase

In this phase, rats were trained to make a different response to the opposite ‘negative’ tone (8 kHz at 65 dB or 2 kHz at 76 dB) to the one that they had heard during the positive training phase. The six ‘Go-pos’ rats that had been trained to shuttle in response to the positive cue were now trained to stay in response to the negative tone (NoGo-neg). The six ‘NoGo-pos’ rats that were previously trained to stay in response to the positive tone were now trained to shuttle in response to the negative tone (Go-neg). As before, each session started with a 120 s ITI followed by the tone sounding for 10 s. For the *NoGo-pos/Go-neg* rats, shuttling during the tone prevented them from receiving a 50 psi air-puff and the tone stopped. If they did not shuttle, they received the air-puff. The *Go-pos/NoGo-neg* rats were required to stay in the current compartment of the shuttle box for the duration of the 10 s tone to avoid the air-puff. If they shuttled to the other compartment during the tone, the tone stopped and they received the air-puff. When an air-puff was presented, all rats were able to escape it by moving to the adjacent chamber. If they did not do this, the air-puff would continue for 10s. Trials on which rats responded correctly were followed by an ITI of 105–115s, and those on which they responded incorrectly were followed by a 115–125 s ITI. During the first two sessions of this phase, six rats were tested per day and the researcher gently guided the rats to make correct responses. From session three onwards, all twelve rats were tested once a day (15 trials per session), five days a week. When rats were performing correctly on more than 65% of trials per session across two consecutive days, they moved on to the final training phase. Training ended after 22 sessions because all *NoGo-pos/Go-neg* rats had failed to achieve criterion at this point, whereas all *Go-pos/NoGo-neg* rats had done so in roughly half the time. *NoGo-pos/Go-neg* rats were moved onto the final discrimination training phase after this point to see whether they were able to learn when both tones were presented together.

##### 2.1.3.3 Discrimination training phase

In this phase, rats were presented with both positive and negative tones during a session. Positive (food) and negative (air-puff) outcomes were delivered contingent on responses exactly as during the first two training phases. Initially rats received 8 positive tone and 8 negative tone trials per session (occasionally 7 of one and 9 of the other due to a software issue) in a pseudorandom order, and the length of ITIs remained the same as during the positive and negative training. No guidance of correct responses was given. After seven sessions (five for one rat), training sessions were altered to comprise 14 reinforced positive trials, 14 reinforced negative trials, 2 non-reinforced positive trials, and 2 non-reinforced negative trials (total of 32 trials per session). This was done so that the rats became accustomed to sessions that were long enough to allow ambiguous cues to be presented as a low proportion (<15%) of total trials in the subsequent ambiguity tests, hence minimising the chances of responses to these non-reinforced cues being extinguished. Partial reinforcement was also implemented to decrease the likelihood of extinction. The ITI was reduced to 60 s regardless of trial outcome. Once rats reached a criterion of more than 70% of trials per session correct for both positive and negative tones over at least two consecutive sessions, ambiguity testing began. Training for *NoGo-pos/Go-neg* rats ended after 14 sessions due to time constraints and because they had shown no sign of learning the Go-neg response during a combined total of 36 sessions in negative tone and discrimination training phases. Given this lack of learning, we investigated whether animals found the air-puff aversive and actively escaped when exposed to it by calculating latency to shuttle out of the compartment in response to the air-puff for the first six negative tone training sessions and the first six discrimination training sessions.

##### 2.1.3.4 Ambiguity tests

During each ambiguity test session, rats were exposed to 16 positive and 16 negative cues (all reinforced) and one of each of five non-reinforced ambiguous cues (3,4,5,6,7 kHz). A random tone order was generated for each ambiguous session. This was the same for all rats. Ambiguous tones were counter-balanced so that the difference in frequency between reference and ambiguous tones was the same across rats on corresponding trials, irrespective of the rats’ tone-reward contingency. For example, if a rat trained with 2 kHz as the positive tone heard a 4 kHz ambiguous tone during trial 11, a rat trained with 8 kHz as the positive tone would also hear an ambiguous tone that differed by 2KHz from its positive reference tone on trial 11 (in this case, 6 kHz). Rats received one ambiguity test session per day, and adjacent tests were separated by a standard discrimination training session on the intermediate day. Most rats performed three tests, but one animal took part in two tests and another in one test. No affect manipulations were made.

### 2.2 Experiment 2: mouse study

The mouse study was carried out in the same way as the rat study using appropriately sized apparatus. The main differences were that condensed milk (a sucrose-rich liquid [65]) was used as the reward rather than sucrose pellets, the order of positive and negative tone training was balanced across subjects, and there were a few small procedural modifications as described below.

#### 2.2.1 Animals and husbandry

The subjects were sixteen male C57BL/6J mice (*Mus musculus*; Harlan UK Ltd, UK). They were six weeks old on arrival and placed in groups of four in standard cages (40 cm L × 25 cm W × 15 cm H, containing sawdust, shredded bedding, red shelter, wooden chew block, two nest-lets and two cardboard tubes), under a 12hr reversed light-dark cycle (lights on 1900-0700). Food (LabDiet) and water were available *ad-libitum.* All mice were checked regularly for any health issues throughout the experiment, which was conducted under UK Home Office licence 30/2954.

#### 2.2.2 Apparatus

Apparatus was the same as for the rat study (see Section 2.1.2) except that two smaller shuttle boxes were used (36 cm L × 25.4 cm W × 30.5 cm H) and automated liquid dippers, rather than food dispensers, were positioned at each end of the shuttle box to provide condensed milk rewards. The copper tubes used to deliver air-puffs were 24.5 cm long (length inside chamber 18 cm), 3 mm in diameter, and perforated with seven × 1 mm diameter holes.

#### 2.2.3 Training and testing

During the first two weeks, the mice were regularly handled using cardboard tunnels and cupped hands, avoiding picking up by the tail [66]. Condensed milk was placed in the home cages during handling sessions to allow animals to become accustomed to the liquid reward. During weeks three and four, the mice were habituated to the shuttle boxes and holding cages used to move animals between rooms. In the rat study, all animals were trained on the positive tone first and it is conceivable that this interfered with subsequent learning about the negative tone and hence was one possible reason for half the rats failing to achieve learning criterion on this latter tone. Consequently, mice were allocated to the same two training contingency groups as used in the rat study (*Go-pos/NoGo-neg* and *NoGo-pos/Go-neg*; N = 8 per group), but half of the animals in each group were trained on the positive tone first and half on the negative tone first. One mouse from each cage was randomly allocated to each of the four contingency/order groups. Apart from this, training procedures were very similar to those used for the rats, and all training and testing was carried out in the dark.

##### 2.2.3.1 Positive tone training

Training tones, contingencies, ITIs, and required Go and NoGo responses were exactly the same as for the rats. When rewarded, the dipper containing condensed milk was available for 10 s. All mice received 16 trials per session. No guidance of correct responses was given. Once the mice performed correctly on more than 70% of trials per session across two consecutive sessions, they moved on to the next training phase.

##### 2.2.3.2 Negative tone training

Negative tone training was identical to that used for rats, except that mice received 16 trials per session. Once the mice performed correctly on more than 70% of trials per session over two consecutive sessions, they moved on to the next training phase. Training ended after 27 sessions due to time constraints.

##### 2.2.3.3 Discrimination training

This phase was virtually identical to that used for the rats except that mice initially received 16 trials per session and once they performed correctly on 70% or more of the trials for both tones, they were then exposed to partial reinforcement sessions comprising 14 reinforced positive trials, 14 reinforced negative trials, 2 non-reinforced positive trials, and 2 non-reinforced negative trials. Once they achieved criterion performance (more than 70% correct on both tones) on this training stage for at least two consecutive sessions, ambiguity testing began. The ITI was 60 s regardless of trial outcome throughout this phase. Training ended after 27 sessions due to time constraints.

##### 2.2.3.4 Ambiguity tests

Each test session was carried out in exactly the same way as for the rats. Due to time constraints, only three of the five *NoGo-pos* mice that achieved criterion during discrimination training were tested on ambiguous cues. They each performed four tests.

### 2.3 Statistical analysis

Data were analysed using IBM SPSS statistics version 23 (IBM Corp. 2014). The number of sessions taken to reach criterion at each training phase was analysed using Kaplen-Meier survival analysis in which each individual either achieved an event on a particular test session (‘criterion performance achieved’) or was censored if it failed to achieve that event by the end of its test sessions. During discrimination training, trials to reach criterion on each tone were analysed separately.

The percentage of correct responses to both cues was calculated for sessions during the discrimination training phase. This was not done for the positive tone and negative tone training sessions because once animals achieved criterion, they moved to the next training phase and hence sample size dropped rapidly preventing meaningful statistical analysis. In discrimination training sessions, because animals had to achieve criterion on *both* tones before moving to ambiguity testing, more data were available (all 12 rats for 14 sessions; 10 mice (4 *Go-pos/NoGo-neg* and 6 *NoGo-pos/Go-neg*) for 8 sessions). Mixed design repeated-measures General Linear Models (GLMs) were used to analyse within-subjects effects of session (polynomial; 14 levels for rats, 8 levels for mice) and between-subjects effects of training contingency group (*Go-pos/NoGo-neg* vs *NoGo-pos/Go-neg*), and their interactions on log-transformed data.

Due to small sample sizes during ambiguity tests and violation of normality assumptions, the proportion of positive responses to cues (i.e. those (Go or NoGo) indicating anticipation of a positive outcome) was analysed using Friedman tests with Monte Carlo estimates of exact p-values based on 10,000 repeated samples of the data.

To investigate whether animals found the air-puff aversive and actively escaped when exposed to it, mixed design repeated-measures GLMs were used to analyse the within-subjects effects of training phase (polynomial; negative tone vs discrimination training phase) and session (polynomial; 6 levels), between-subjects effects of training contingency group (*Go-pos/NoGo-neg* vs *NoGo-pos/Go-neg*), and their interactions, on these data.

To determine baseline rates of movement between the two chambers of the shuttle box, mean shuttle rate/min during the 60 s ITIs of the last 5 discrimination training sessions before criterion was achieved (or before training was stopped for those animals that did not achieve criterion) were calculated for all animals. Mixed design repeated-measures GLMs were used to analyse the within-subjects effects of session (polynomial; 5 levels), between-subjects effects of training contingency *Go-pos/NoGo-neg* vs *NoGo-pos/Go-neg*), and their interactions. Rat data were log transformed but studentized residuals fulfilled normality assumptions for mouse data.

Statistical significance was set at p < 0.05, acknowledging that this threshold is a pragmatic convention for reporting results that does not on its own indicate the size or importance of a result [67] and that, although comparisons were pre-planned, the study was exploratory in nature. All p values are reported. Sample sizes for analyses are shown in figure legends.

## 3 Results

Results are organised according to different behavioural measures and, under each heading, findings from each species are presented separately.

### 3.1 Number of sessions to achieve learning criterion in each training phase

#### 3.1.1 Rats

**Fig. 2:**
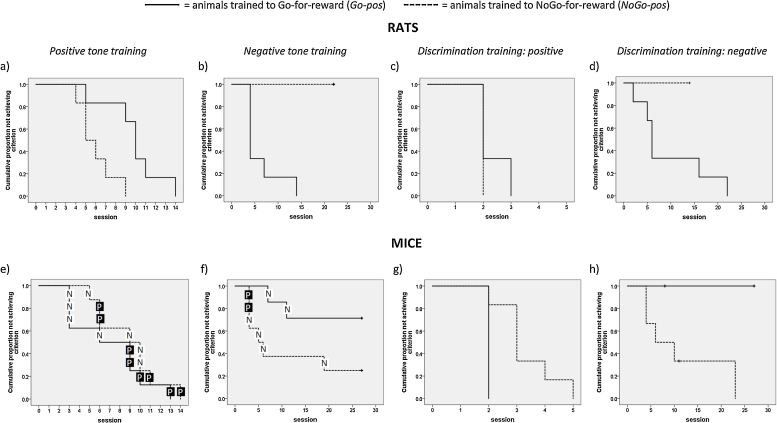
Kaplan-Meier survival curves showing the cumulative proportion of subjects not achieving criterion performance across training sessions for rats during (a) the positive tone training phase, (b) the negative tone training phase, and for the (c) positive and (d) negative tones during discrimination training (N = 6 *Go-pos/NoGo-neg* and 6 *NoGo-pos/Go-neg* rats). The corresponding data are shown for mice in panels (e)–(h) respectively ((e) N = 8 *Go-pos/NoGo-neg* and 8 *NoGo-pos/Go-neg* mice; (f) N = 7*Go-pos/NoGo-neg* and 8 *NoGo-pos/Go-neg* mice; (g,h) N = 4 *Go-pos/NoGo-neg* and 6 *NoGo-pos/Go-neg* mice). Animals trained on the *Go-pos* task are shown by solid lines, and those trained on the *NoGo-pos* task by dashed lines. Vertical ticks on curves indicate subjects that are censored due to finishing the experiment without achieving learning criterion. This happened for three mice prior to the end of training in the discrimination training phase because they failed to reach learning criterion on the negative tone after the time limit for this study (a total of c.40 training sessions) had elapsed (h). To allow ease of comparison, the x and y-axis scales are the same for rats and mice within each training category. In e and f, the training order group of each mouse that achieves criterion is shown (P = trained on positive tone first; N = trained on negative tone first).

All rats achieved learning criterion by session 14 of the positive tone training phase, and rats in the *NoGo-pos/Go-neg* training group (hereafter *NoGo-pos*) reached it significantly earlier (Log-Rank chi-square = 6.56, df = 1, p = 0.01; Fig. 2a) than those in the *Go-pos/NoGo-neg* group (hereafter *Go-pos*). However, during the negative training phase, all *Go-pos* rats reached criterion by session 14, whilst none of the rats in the *NoGo-pos* reached criterion even after 22 sessions (Log-Rank chi-square = 12.54, df = 1, p < 0.001; Fig. 2b). These analyses include some sessions during which rats were occasionally guided to perform the correct response. The number of such sessions differed significantly between groups during the negative training phase (*Go-pos*: 2 (1–2); *NoGo-pos*: 7 (6–7); Mann-Whitney U = 0, N = 12, p = 0.002), and there was some suggestion of a difference during positive training (*Go-pos*: median = 2 (range = 1–3); *NoGo-pos*: 1 (1); Mann-Whitney U = 30, N = 12, p = 0.065). In both cases more guidance was given when training active responses (Go-neg and Go-pos). Removal of negative training phase sessions in which assistance was received had no effect on survival analysis results because all *NoGo-pos* rats failed to learn to shuttle to avoid the air pulse.

**Fig. 3:**
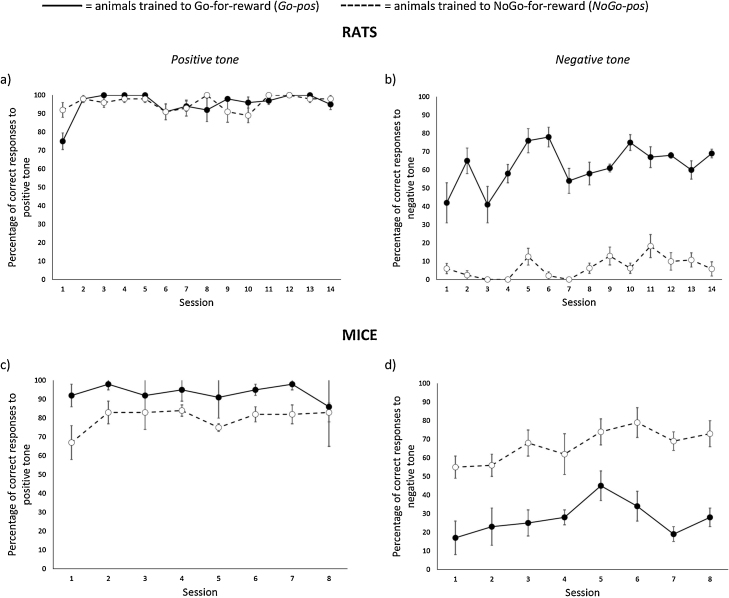
Graphs showing the percentage of correct responses made by rats to (a) the positive tone and (b) the negative tone during discrimination training sessions (N = 6 *Go-pos/NoGo-neg* and 6 *NoGo-pos/Go-neg* rats). The corresponding data are shown for mice in panels (c) and (d) respectively (N = 4 *Go-pos/NoGo-neg* and 6 *NoGo-pos/Go-neg* mice). Animals trained on the *Go-pos* task are shown by solid lines and filled circles, and those trained on the *NoGo-pos* task by dashed lines and open circles. Data are means ± 1 s.e.m.

Despite not achieving criterion during the negative training phase, *NoGo-pos* rats were moved on to discrimination training in case the combination of both positive and negative cues during this phase facilitated learning. During discrimination training, both groups reached criterion on the positive cue within 3 sessions (Log-Rank chi-square = 2.2, df = 1, p = 0.14; Fig. 2c). However, as during negative training, *Go-pos* rats performed better in response to the negative cue than *NoGo-pos* rats (Log-Rank chi-square = 5.45, df = 1, p = 0.02; Fig. 2d). The latter continued to show very poor performance across 14 training sessions (Fig. 3b) at which point their training was stopped. In contrast, 4 out of 6 *Go-pos* rats achieved criterion by session 14, and the remaining two achieved criterion at sessions 16 and 22 (Fig. 2d).

#### 3.1.2 Mice

All mice achieved criterion performance by session 14 of the positive tone training phase and there was no significant difference between *Go-pos* and *NoGo-pos* animals (Log-Rank chi-square = 0.99, df = 1, p = 0.32; Fig. 2e). During the negative training phase, one *Go-pos* animal died and only 2 out of the remaining 7 reached the learning criterion by session 27, whilst 6 out of 8 *NoGo-pos* animals did (Log-Rank chi-square = 3.83, df = 1, p = 0.05; Fig. 2f). Because half the mice were trained on the positive tone first and half on the negative tone first, we analysed the effects of training order on learning of the four responses. Mice trained on the negative tone first learnt the Go-pos response faster than those trained on the positive tone first (Log-Rank chi-square = 7.6, df = 1, p = 0.006; Fig. 1e), but there were no other effects of training order (NoGo-pos: Log-Rank chi-square = 0.88, df = 1, p = 0.35; Go-neg: Log-Rank chi-square = 0.91, df = 1, p = 0.34; NoGo-neg: Log-Rank chi-square = 1.74, df = 1, p = 0.19; Fig. 2e and f).

The 8 successful learners moved on to the discrimination training phase together with two of the *Go-pos* animals who failed to reach criterion on the negative tone but subsequently learnt the positive response very fast. All animals achieved criterion on the positive tone after 5 sessions, with *Go-pos* animals learning significantly faster than *NoGo-pos* animals (Log-Rank chi-square = 6.0, df = 1, p = 0.014; Fig. 2g). However, *NoGo-pos* mice reached criterion on the negative tone significantly earlier than *Go-pos* animals, all four of whom failed to achieve criterion (Log-Rank chi-square = 4.92, df = 1, p = 0.027; Fig. 2h). Training contingency thus had effects on mice that were opposite to those observed for rats.

### 3.2 Percentage correct responses to positive and negative tones during discrimination training

#### 3.2.1 Rats

During the first 14 discrimination training sessions, performance on the positive tone was affected by a training contingency * session interaction (F_13,130_ = 2.23, p = 0.011; Fig. 3a) and by session (F_13,130_ = 4.46, p < 0.001), but not by training contingency (F_1,10_ = 0.28, p = 0.61). The interaction indicated a different temporal pattern in the two groups, with a more marked increase in performance from session 1–2 in the *Go-pos* group. For the negative tone, there were significant effects of contingency * session (F_13,130_ = 2.12, p = 0.017; Fig. 3b), contingency (F_1,10_ = 406.2, p < 0.001), and session (F_13,130_ = 3.11, p < 0.001). The interaction effect is likely due to different temporal patterning of the performance of *Go-pos* and *NoGo-pos* rats across time, and it is clear that the former performed better than the latter.

#### 3.2.2 Mice

During the first eight discrimination training sessions, *Go-pos* mice performed better than *NoGo-pos* mice on the positive tone (F_1,8_ = 6.5, p = 0.034; Fig. 3c), but there were no statistically significant effects of session (F_7,56_ = 1.23, p = 0.30) or contingency * session (F_7,56_ = 0.98, p = 0.45). Conversely, *NoGo-pos* mice performed better than *Go-pos* mice on the negative tone (F_1,8_ = 16.40, p = 0.004; Fig. 3d), and there was a statistically significant effect of session (F_7,56_ = 3.78, p = 0.002) but not of contingency * session (F_7,56_ = 1.70, p = 0.13). The session effect may reflect the pattern seen in both groups of a gradual increase in performance across sessions 1–5 followed by a dip on session 7 and an increase on session 8.

### 3.3 Ambiguity tests

#### 3.3.1 Rats

**Fig. 4:**
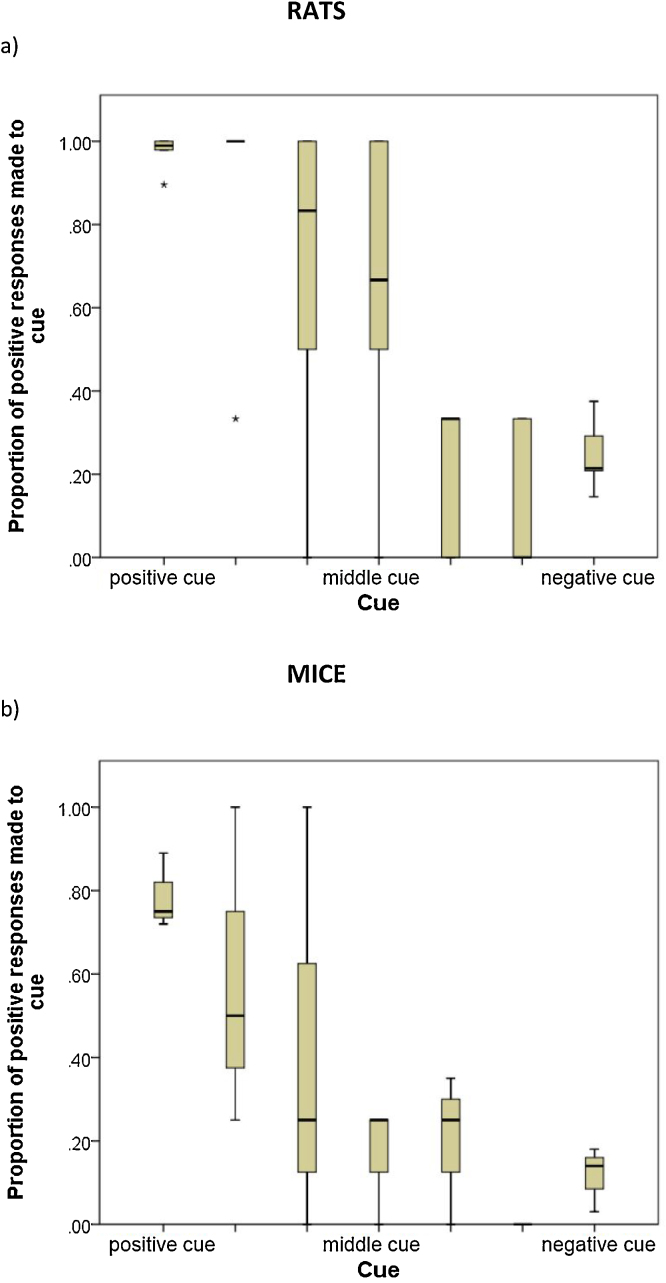
Boxplots showing the proportion of positive responses made to tones during ambiguity tests by (a) rats (N = 6) and (b) mice (N = 3). The positive and negative training tones are 2 and 8 kHz (counterbalanced across subjects), and the intermediate tones are 3,4,5,6,7 kHz. Boxplots show medians, quartiles and ranges. Asterisks indicate points that are more than 3 inter-quartile ranges away from the upper or lower quartile.

Because none of the *NoGo-pos* rats achieved criterion performance during discrimination training, only animals in the *Go-pos* group were tested on ambiguous cues. Across the three test sessions, rats showed the expected generalisation response across cues (Friedman Test: chi-square = 22.72, df = 6, Monte Carlo estimated p < 0.001; Fig. 4a), performing proportionally more positive (Go) responses to cues close to the positive tone than to cues nearer the negative tone. Although median values across the five ambiguous cues indicate a continuous change in the proportion of positive responses, we cannot conclude linearity from this small sample.

#### 3.3.2 Mice

None of the *Go-pos* mice achieved criterion during discrimination training. Across the four ambiguity tests, the three *NoGo-pos* mice that were tested showed a greater proportion of positive (NoGo) responses to tones close to the positive training tone than to those close to the negative training tone, indicating the expected generalisation of responses across cues (Friedman Test: chi-square = 13.02, df = 3, Monte Carlo estimated p = 011; Fig. 4b).

### 3.4 Escape from air-puff

#### 3.4.1 Rats

**Fig. 5:**
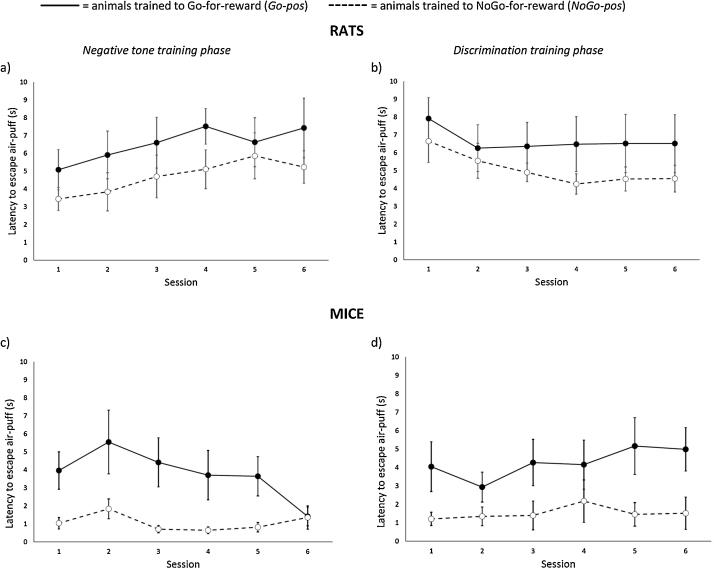
Graphs showing the latency to escape air-puffs (s) after these were delivered following an incorrect response to a negative tone for rats during the first 6 sessions of the (a) negative tone training phase and (b) discrimination training phase (N = 5 *Go-pos/NoGo-neg* and 6 *NoGo-pos/Go-neg* rats). The corresponding data are shown for mice in panels (c) and (d) respectively (N = 4 *Go-pos/NoGo-neg* and 4 *NoGo-pos/Go-neg* mice). Animals trained on the *Go-pos* task are shown by solid lines and filled circles, and those trained on the *NoGo-pos* task by dashed lines and open circles. Data are means ± 1 s.e.m.

Although rats in the *NoGo-pos* group failed to achieve criterion on the ‘Go to avoid air-puff’ response to the negative tone, they did not differ significantly from *Go-pos* rats in their latency to escape the 10 s air-puff when it was delivered to them after an incorrect response to the negative cue (F_1,9_ = 1.59, p = 0.24). A significant training phase * session interaction (F_5,45_ = 6.76, p < 0.001; Fig. 5a and b) indicated that escape latencies tended to increase during the first 6 negative training sessions but decrease or remain stable during the first 6 discrimination training sessions. There were no statistically significant effects of training phase (F_1,9_ = 0.76, p = 0.40) and session (F_5,45_ = 1.13, p = 0.36), and no contingency * training phase (F_1,9_ = 0.04, p = 0.84), contingency * session (F_5,45_ = 1.15, p = 0.35), or contingency * training phase * session (F_5,45_ = 0.65, p = 0.66) interactions.

#### 3.4.2 Mice

As with rats, mice from both training contingency groups escaped the air-puff during the 10 s that it was delivered to them following an incorrect response. Significant training contingency * training phase * session (F_5,30_ = 3.56, p = 0.012; Fig. 5c and d), and training phase * session (F_5,30_ = 5.2, p = 0.001) interactions, and a near significant effect of contingency (F_1,6_ = 5.72, p = 0.054) indicated that *Go-pos* mice were slower to escape the air-puff, and their escape latencies tended to decrease across the first 6 negative training sessions but increase across the first 6 discrimination training sessions, whilst the escape latencies of *NoGo-pos* mice remained consistently fast across both sessions. There were no significant effects of training phase (F_1,6_ = 5.084, p = 0.32) or session (F_5,30_ = 0.54, p = 0.74), and no contingency * training phase (F_1,6_ = 0.001, p = 0.84) or contingency * session (F_5,30_ = 1.07, p = 0.40) interactions

### 3.5 Baseline shuttle rates

#### 3.5.1 Rats

**Fig. 6:**
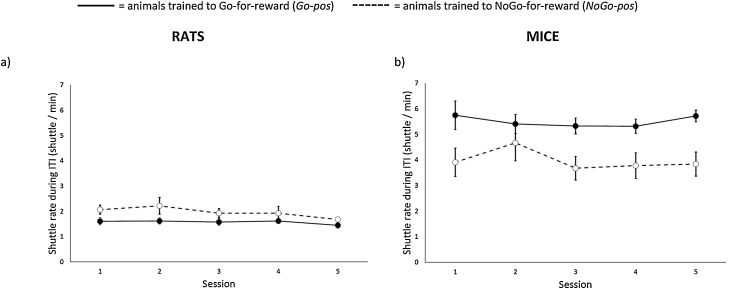
Graphs showing shuttle rate (/min) during the 60 s inter-trial-intervals of the last 5 discrimination training sessions for subjects trained on (a) rats (N = 6 *Go-pos/NoGo-neg* and 6 *NoGo-pos/Go-neg* rats) and (b) mice (N = 4 *Go-pos/NoGo-neg* and 6 *NoGo-pos/Go-neg* mice). Animals trained on the *Go-pos* task are shown by solid lines and filled circles, and those trained on the *NoGo-pos* task by dashed lines and open circles. Data are means ± 1 s.e.m.

There was a statistically significant effect of session (F_4,40_ = 3.10, P = 0.026; Fig. 6a) on mean shuttle rate of rats during the 60 s ITIs of the last 5 discrimination training sessions, likely reflecting a small decrease in shuttle rate at the end of this phase. Contingency (F_1,10_ = 3.55, P = 0.089) and contingency * session effects (F_4,40_ = 0.69, P = 0.60) were not significant.

#### 3.5.2 Mice

Animals in the *Go-pos* training group shuttled more than those in the NoGo pos group (F_1,8_ = 5.42, P = 0.048; Fig. 6b), and shuttle rates were not significantly affected by session (F_4,32_ = 1.38, P = 0.26) or a contingency * session interaction (F_4,32_ = 1.53, P = 0.22).

## 4 Discussion

The aim of this study was to develop a counter-balanced Go/NoGo judgement bias task for laboratory rats and mice, in order to allow investigation of the potential effects of Pavlovian control on instrumental performance in the task. For the rat (Sprague Dawley) and mouse (C57BL/6J) strains used, one of the two counter-balanced contingencies in the shuttle box task was learnt but the other was not. Strikingly, this differed between species/strains with rat data supporting the hypothesis that Pavlovian control favours *Go-pos/NoGo-neg* learning whilst mouse data supported the opposite − that it favours *Go-neg*/*NoGo-pos* learning. Because of the failure of both species to fully learn both contingencies, successful development of a completely counter-balanced Go/NoGo judgement bias task was not achieved in this study.

For the training contingency groups that reached criterion during discrimination sessions, responses during ambiguity tests showed the expected generalisation of Go and NoGo responses across tones of intermediate frequency to the positive and negative cues, thus indicating that the task had been learnt by both rats and mice. This adds to the successful use of tone cues in rat judgement bias tasks [1], [15], [17], [64], and provides the first demonstration that mice can also learn a tone-based discrimination in this context. Speed of learning compared favourably with other automated judgement bias tasks in rats, with median sessions to criterion across all three phases in the learnt contingency (6 rats) totalling 24.5, including assisted sessions, compared to 27 for reward–reward tasks [19] and up to 60 for reward-punishment tasks [15], [64], [68]. For mice, the corresponding median number of sessions was 22.5 (5 mice). With successful training of the opposite contingencies, the task could therefore be used to investigate the influence of affect manipulations, and to test the hypothesis that positive affect may enhance vigour and activity and thus interfere with outcome-focused instrumental decisions differently in *Go-pos* and *NoGo-pos* tasks [59].

Our most notable finding was that each species/strain learnt only one of the contingencies. In line with the prediction that Pavlovian controllers favour active approach responses in a rewarding context and inhibition or withdrawal in a punishing context [54], [55], [57], [58], Sprague-Dawley rats achieved learning criterion on the *Go-pos*/*NoGo-neg* task, but failed to do so on the reverse contingency task. However, for C57BL/6J mice, completely the opposite was found. Most mice were able to learn the *NoGo-pos*/*Go-neg* task, but none learnt the reverse contingency. These findings support the interpretation that in Sprague-Dawley rats, as in humans [58], Pavlovian control interfered with instrumental learning in the *NoGo-pos* contingency group, thus accounting for their failure to achieve learning criterion. In contrast, it could be argued that Pavlovian control in C57BL/6J mice interfered with learning in the *Go-pos* group, favouring NoGo-for-reward and Go-to-avoid-punishment responses. This is conceivable in a largely herbivorous prey species where food tends to be static and predators active, in contrast to the opportunistically omnivorous rat where moving food is part of the natural diet. If correct, this indicates that Pavlovian predispositions may vary across species in an ecologically consonant way.

There is a difference between the findings of Guitart-Masip et al. [58] and those reported here. Humans trained on an orthogonalised Go/NoGo task performed better when required to make ‘Go-to-win’ rather than ‘NoGo-to-win’ responses, and when required to make ‘NoGo-to-avoid-losing’ rather than ‘Go-to-avoid-losing’ responses [54], [58]. In both rats and mice, on the other hand, animals were able to learn both Go and NoGo responses when faced with cues predicting reward, but not in response to cues predicting punishment; failure to achieve learning criterion was restricted solely to cues signalling a negative outcome in rats required to make a Go response to these cues, and mice required to make a NoGo response.

One potential explanation for this finding is that, in addition to Pavlovian linkages between valence and vigour (e.g. enhanced activity in anticipation of reward [54]), a ‘baseline’ activity rate dictates the ease with which active and passive responses can be implemented during learning. Fig. 6 shows that rats and mice clearly differed in levels of baseline activity measured as shuttle rate during inter-trial intervals; mice shuttled two to three times as frequently as rats. Species differences in behavioural ecology may underlie this difference (e.g. mice needing to move rapidly when outside burrows in order to minimise predation risk), and support the notion that we detected species-typical differences in task performance rather than strain-specific ones, although we cannot completely rule out the latter possibility. The idea that mice showed a predominantly ‘Go’ baseline response relative to rats who favoured less movement (NoGo) also appears to be supported by data from reward learning where both species were able to learn both contingencies; mice learnt ‘Go-for-reward’ quicker than ‘NoGo-for-reward’ during discrimination training (Fig. 2g), and the opposite was true for rats during positive tone training (Fig. 2a).

Following this line of argument, our findings indicate that both species were able to over-ride their favoured baseline response in order to acquire food (i.e. rats were able to Go for food and mice to NoGo for food), but not to avoid the air-puff. This might indicate that the implementation of Pavlovian control in each species involves suppression of baseline actions (and/or activation of contrasting actions) in response to reward prediction, and the expression of baseline actions in response to punishment prediction. This has some similarities with the notion that activities directed at avoiding threat or danger should be pre-eminent over, and more readily expressed than, those directed at acquiring reward, because the costs of failing to make the appropriate response in the former context are more severe [69].

Initial training of responses to positive and negative tones was balanced for order across subjects in the mouse study in case training positive tones first (as is common practice in comparable tasks [15], [17], [64]) interfered with learning about negative tones in the rat study. However, there was no evidence that training order influenced the ease with which mice learnt both Go and NoGo responses to the negative tone, although training responses to the negative tone first did appear to speed up learning of the Go-pos response. The reason for this latter finding is unclear.

From a practical perspective, full implementation of a counter-balanced Go/NoGo judgement bias task would require use of a negative stimulus that could motivate appropriate instrumental avoidance responses in opposition to Pavlovian predispositions and baseline activity. Although the 50 psi air puff stimulus used here was unable to do this, it was sufficiently aversive to motivate *escape* behaviour in rats and mice from both training contingencies during the 10 s that it was presented following an incorrect response to a negative tone. Interestingly, *NoGo-neg* mice were slower to escape the air-puff than *Go-neg* mice. This may have been because *NoGo-neg* mice received an air-puff immediately after they (incorrectly) shuttled (Go action) during a negative tone, whilst *Go-neg* mice received an air-puff if they failed to shuttle. If the probability that a mouse performs a Go action (shuttle) increases with time since the last one was performed this could explain the observed differences in escape latencies. Another possible explanation is that escape behaviour was consistent with trained avoidance behaviour in the *Go-neg* mice but not the *NoGo-neg* mice, hence resulting in faster responses in the former animals.

On the basis of these observations, it seems likely that employing a more potent stimulus would allow full training of a counter-balanced task in both species. To minimise stress in the testing context, which is important in tasks designed to assess affective states and also for 3Rs *Refinement* reasons, the preferred option would be a more powerful, but non-painful, air-puff (e.g. mice can learn to inhibit responses to avoid an air puff [70]), and we are currently developing related tasks that can successfully use air-puff in this context.

## 5 Conclusions

A deeper understanding of the learning processes mediating performance in judgement bias tasks may aid in their interpretation, and shed light on contradictory results in the literature. One potentially important influence is that of Pavlovian control on instrumental responses in Go/NoGo tasks. To investigate this, we implemented a counter-balanced Go/NoGo task and found that Sprague Dawley rats readily learnt Go-for-reward/NoGo-to-avoid-punishment responses but failed to learn the reverse, whilst C57BL/6J mice did exactly the opposite. This implies that Pavlovian predispositions may vary between species/strain, perhaps reflecting differences in foraging and predator avoidance ecology and/or in baseline activity rates. The use of a more powerful air-puff may enable both species to learn both contingencies, thus providing a task that offers advantages over existing methods in terms of speed of training, implementation using widely available automated equipment, a decreased susceptibility to confounding interpretations, and the opportunity to unpick the influences of Pavlovian control on decisions in commonly used unbalanced Go-for-reward/NoGo-to-avoid-punishment tests. The 3Rs implications will be to facilitate more widespread use of this approach to assessing animal welfare, which has been limited by the time demands of training procedures, and hence to allow better detection of animal welfare problems and assessment of the effectiveness of refinements to improve welfare.
